# Revisiting the association between skin toxicity and better response in advanced cancer patients treated with immune checkpoint inhibitors

**DOI:** 10.1186/s12967-020-02612-5

**Published:** 2020-11-11

**Authors:** Nicholas Gulati, Douglas Donnelly, Yingzhi Qian, Una Moran, Paul Johannet, Judy Zhong, Iman Osman

**Affiliations:** 1 The Ronald O. Perelman Department of Dermatology, New York University Grossman School of Medicine, New York, NY USA; 2 Department of Population Health, New York University Grossman School of Medicine, New York, NY USA

**Keywords:** Melanoma, Immune checkpoint inhibition, Immune-related adverse events, Skin toxicity, Advanced cancer

## Abstract

**Background:**

Immune checkpoint inhibition (ICI) improves survival outcomes for patients with several types of cancer including metastatic melanoma (MM), but serious immune-related adverse events requiring intervention with immunosuppressive medications occur in a subset of patients. Skin toxicity (ST) has been reported to be associated with better response to ICI. However, understudied factors, such as ST severity and potential survivor bias, may influence the strength of these observed associations.

**Methods:**

To examine the potential confounding impact of such variables, we analyzed advanced cancer patients enrolled prospectively in a clinicopathological database with protocol-driven follow up and treated with ICI. We tested the associations between developing ST, stratified as no (n = 617), mild (n = 191), and severe (n = 63), and progression-free survival (PFS) and overall survival (OS) in univariable and multivariable analyses. We defined severe ST as a skin event that required treatment with systemic corticosteroids. To account for the possibility of longer survival associating with adverse events instead of the reverse, we treated ST as a time-dependent covariate in an adjusted model.

**Results:**

Both mild and severe ST were significantly associated with improved PFS and OS (all P < 0.001). However, when adjusting for the time from treatment initiation to time of skin event, severe ST was not associated with PFS benefit both in univariable and multivariable analyses (P = 0.729 and P = 0.711, respectively). Receiving systemic steroids for ST did not lead to significant differences in PFS or OS compared to patients who did not receive systemic steroids.

**Conclusions:**

Our data reveal the influence of time to event and its severity as covariates in analyzing the relationship between ST and ICI outcomes. These differences in outcomes cannot be solely explained by the use of immunosuppressive medications, and thus highlight the importance of host- and disease-intrinsic factors in determining ICI response and toxicity.

Trial registration: The patient data used in this manuscript come from patients who were prospectively enrolled in two institutional review board-approved databases at NYU Langone Health (institutional review board #10362 and #S16-00122).

## Background

Immune checkpoint inhibition (ICI) causes tumor regression in patients with several types of cancer including metastatic melanoma (MM) by introducing monoclonal antibodies that activate the adaptive immune system [1–3]. This non-specific immune activation frequently leads to transient or chronic immune-related adverse events (irAEs), which recent data suggest is attributed to an interplay of host-specific factors [4–7]. Typically presenting as pruritus and/or rash, skin toxicity (ST) is the most common ICI-induced toxicity, and several reports have associated its development with improved clinical outcomes in melanoma patients [8–13]. However, these studies were limited by understudying possible confounding variables that may influence or offer insight into these observed associations.

First, previous studies were not adequately powered to evaluate differences based on the severity of the ST. The Common Terminology Criteria for Adverse Events (CTCAE) does not account for steroidal intervention, a critical patient care branch point, in the severity grading scale for rash. Additionally, subjects who progress or die shortly after treatment initiation have no opportunity to experience ST, which introduces potential bias in analyses that do not account for this fact. Such shortcomings challenge the notion that developing ST more likely portends a positive outcome.

We here investigated the association between ST and ICI outcomes using a clinically relevant grading system to assess more granularly the relationship according to severity. Further, we tested the possibility that survivor bias may reduce the strength of the perceived relationship in patients treated for various advanced cancers.

## Methods

We analyzed the relationship between ST and progression-free survival (PFS) and overall survival (OS) in a cohort of 673 advanced cancer patients receiving ICI, enrolled and prospectively followed up in two New York University Langone Health databases. The cancer types included were: melanoma, brain, breast, genitourinary, head and neck, kidney, liver and intrahepatic bile ducts, lung, mesothelial and soft tissue, ovarian and fallopian tube, pancreatic, non-melanoma skin, stomach, and uterine. Stage III-IV patients treated with ICI from August 2012 through January 2020 were classified according to the development of no, mild, and severe ST. Patients’ best response was evaluated according to response evaluation criteria in solid tumors (RECIST), and data were recorded as complete response, partial response, stable disease, mixed response, and progression of disease. Overall response rate (ORR) was calculated in patients treated for metastatic disease by dividing the sum of treatment lines with complete response, partial response, and stable disease from the total number of treatment lines analyzed in each of the three ST categories.

In assessing the cause of ST, the patient and/or physician reported the event, which was recorded in the electronic medical record, and an immunologist-dermatologist independently confirmed the event was ICI-related. Mild ST was defined as a skin adverse event that did not require treatment with systemic corticosteroids, and was successfully managed with topical corticosteroids, topical antipruritics, oral antipruritics, and/or observation. Severe ST was defined as a skin adverse event that prompted the treating oncologist to employ systemic corticosteroids, specifically for the ST. Other site-specific toxicities were recorded using the CTCAE v5.0, and included gastrointestinal, hepatic, endocrine, neural, and other. We assessed the temporal relationship between other site-specific toxicities and ST, as well as the effects of concomitance on survival outcomes.

Given their immunosuppressive functions, we investigated the effects of systemic corticosteroids administered to the patient during each treatment line on survival outcomes. The medication history was recorded from the date of the first ICI treatment to the date of the response noted. The highest dose of prednisone, dexamethasone, and methylprednisolone administered for any clinical indication was recorded. The doses were normalized to prednisone, with a conversion of 6.7 × for dexamethasone and 1.3 × for methylprednisolone.

### Statistical analysis

Baseline patient characteristics in the cohort and ORR were compared among the three ST categories using the Chi square test. Kaplan–Meier curves were generated and compared by the log-rank test to estimate OS and PFS distribution for each ST group. Using univariable and multivariable cox proportional hazard models, we analyzed the associations between ST and PFS/OS. The multivariable analysis, which was stratified by cancer type, adjusted for age, gender, number of metastatic sites, stage, and Eastern Cooperative Oncology Group (ECOG) score at treatment initiation.

To account for potential survivor bias, we treated ST as a time-dependent variable. In doing so, we altered the ST covariate to adjust for the time from treatment initiation to the time of the skin adverse event in assessing survival outcomes. The cox model was fitted by considering the ST covariate of 0 before the irAE onset and 1 after the irAE onset, as previously described [14].

## Results

Table 1 illustrates the baseline characteristics of the cohort of 673 advanced cancer patients. Of the 871 treatment lines analyzed, severe ST was observed in 63 (7.2%), mild ST in 191 (22.0%), and no ST in 617 (70.8%) (Table 2). MM patients were significantly more likely to experience severe ST than patients with other advanced cancers. Pruritus and rash were the most common ST, appearing in 192 (75.3%) and 188 (73.7%) of the treatment lines with ST, respectively. Other ST included vitiligo (n = 7), facial erythema (n = 1), psoriasiform eruption (n = 4), lichen planus (n = 2), dermatomyositis (n = 1), alopecia (n = 2), bullous pemphigoid (n = 1), and worsening of scleroderma (n = 1).

**Table 1:** Patient characteristics (n = 673**)**

Characteristic	N (%)
Age (mean(SD))	63.4 (13.6)
Sex	
Male	375 (55.7)
Female	298 (44.3)
Ethnicity	
Non-Hispanic white	490 (72.8)
Non-Hispanic Black	49 (7.3)
Non-Hispanic Asian Pacific Islander	63 (9.4)
Hispanic	57 (8.5)
Other/Unknown	14 (2.1)
Stage at initiation	
Stage III	139 (20.7)
Stage IV	531 (79.3)
ECOG at initiation	
0	312 (47.9)
> 1	340 (52.1)
Months of follow up from initiation (median (range))	18.7 (1.2–77.9)
Lines of treatment (median(range))	1 (1–7)

**Table 2:** Treatment line characteristics (n = 871), grouped by no ST (None), mild ST, and severe ST

	**None**	**Mild**	**Severe**	**P**
n (%)	617 (70.8)	191 (22.0)	63 (7.2)	
Cancer (%)				
Melanoma	257 (41.7)	86 (45.0)	44 (69.8)	<0.001
Brain	20 (3.2)	3 (1.6)	1 (1.6)	
Breast	31 (5.0)	7 (3.7)	0 (0.0)	
Genitourinary	17 (2.8)	9 (4.7)	0 (0.0)	
Head and neck	25 (4.1)	4 (2.1)	0 (0.0)	
Kidney	26 (4.2)	15 (7.9)	6 (9.5)	
Liver and intrahepatic bile ducts	25 (4.1)	7 (3.7)	2 (3.2)	
Lung	131 (21.2)	47 (24.6)	6 (9.5)	
Mesothelial and soft tissue	26 (4.2)	0 (0.0)	0 (0.0)	
Ovarian and fallopian tube	13 (2.1)	5 (2.6)	1 (1.6)	
Pancreatic	8 (1.3)	1 (0.5)	1 (1.6)	
Non-melanoma skin	23 (3.7)	0 (0.0)	0 (0.0)	
Stomach	9 (1.5)	4 (2.1)	1 (1.6)	
Uterine	6 (1.0)	3 (1.6)	1 (1.6)	
ICI treatment category (%)				0.017
Anti-CTLA-4	63 (10.2)	23 (12.0)	17 (27.0)	
Anti-CTLA-4 + Anti-PD-1	123 (19.9)	39 (20.4)	14 (22.2)	
Anti-PD-1	402 (65.2)	122 (63.9)	31 (49.2)	
Anti-PD-L1	25 (4.1)	7 (3.7)	1 (1.6)	
Other	4 (0.6)	0 (0.0)	0 (0.0)	
Adjuvant vs metastatic (%)				0.301
Adjuvant	44 (7.1)	21 (11.0)	8 (12.7)	
Metastatic	572 (92.7)	171 (89.0)	55 (87.3)	
Neo-adjuvant	1 (0.2)	0 (0.0)	0 (0.0)	
Response to treatment (%)				<0.001
Complete response	68 (11.4)	41 (21.7)	8 (13.1)	
Partial response	104 (17.4)	57 (30.2)	17 (27.9)	
Stable disease	99 (16.5)	37 (19.6)	13 (21.3)	
Progression of disease	328 (54.8)	54 (28.6)	23 (37.7)	
Hospitalization due to toxicity = Yes (%)	57 (9.2)	13 (6.8)	4 (6.3)	0.469
Discontinued due to toxicity = Yes (%)	87 (14.1)	21 (11.0)	18 (28.6)	0.002
Discontinued due to progression of disease = Yes (%)	409 (66.3)	84 (44.0)	29 (46.0)	<0.001

**Fig. 1:**
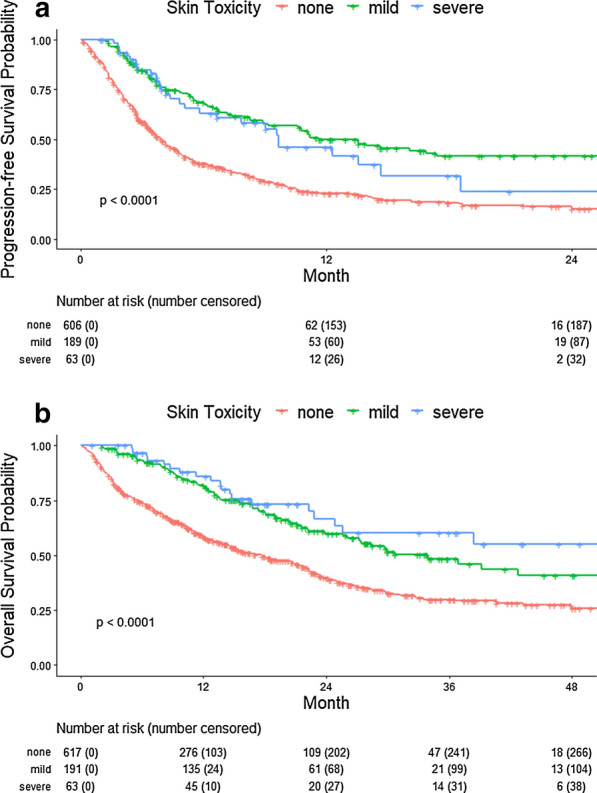
Mild and severe skin toxicity (ST) are both associated with improved PFS and OS in advanced cancer patients treated with ICI. **a** Progression-free survival (PFS) and **b** overall survival (OS) by ST grouping. All p-values are from the log-rank tests

Compared to none, development of mild ST was statistically significantly associated with improved PFS (hazard ratio [HR] = 0.39 [0.31, 0.49], P < 0.001) and OS (HR = 0.50 [0.39, 0.63], P < 0.001). Similarly, compared to none, development of severe ST was statistically significantly associated with improved PFS (HR = 0.52 [0.36, 0.73], P < 0.001) and OS (HR = 0.46 [0.30, 0.71], P < 0.001) (Fig. 1). Among melanoma treatment lines for metastatic disease (326/387), mild ST was also statistically significantly associated with better response compared with no ST (ORR = 63.4% vs 42.5%, P = 0.003). After adjusting for age, gender, number of metastatic sites, stage, and ECOG score at treatment initiation, development of both mild and severe ST remained statistically significantly associated with both improved PFS and OS, compared to no ST (all P < 0.001).

**Fig. 2:**
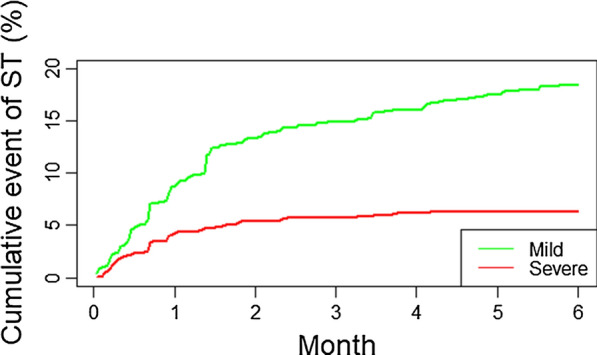
Cumulative hazard plot of mild and severe ST with time. Mild ST (green) and severe ST (red) cumulative incidences plotted over time. Most ST events were noted to occur within three months of starting ICI

ST was significantly associated with the development of other toxicities, including gastrointestinal (P = 0.033), hepatic (P = 0.008), and endocrine (P < 0.001). In order to mitigate potential survivor bias, we treated ST as a time-dependent covariate. By plotting cumulative event of ST with time, more mild ST events occurred with time as compared to severe ST. Most ST events were noted to occur within three months of starting ICI (Fig. 2). After adjusting for the time from treatment initiation to time of skin event, the association of mild ST with PFS and OS maintained statistical significance in multivariable analysis (P = 0.023 and P = 0.001, respectively). On the other hand, severe ST maintained statistical significance for OS (P = 0.037), but lost statistical significance for PFS (P = 0.711).

## Discussion

Our study revisits the link between response and toxicity to ICI by evaluating the most common organ-specific group of irAEs, ST. The results suggest that a threshold exists for the perceived clinical benefit associated with ICI-induced ST, which may result from prolonged treatment with ICI leading to greater potential for an adverse event. While elucidating the mechanisms that bridge toxicity and immunotherapy outcomes requires extensive investigation, this analysis provides a more granular exploration of reported positive data.

First, utilizing a new definition of severe ST allowed this study to have sufficient events for a more nuanced analysis of the association of ST with clinical benefit. Clinical trial data report severe ST in 1.6–5.8% of treatments [1, 2], but our study found severe ST in 7.2% of treatment lines. While this may appear to overstate the burden of severe ST, 25.2–43.2% of patients in the CheckMate 067 trial were treated for their ST with immunomodulatory medication, including both topical and systemic immunosuppressive agents. Thus, clinical management decisions in clinical trials, where adverse event reporting is highest, support the utility of this new definition. Also, our definition of mild skin toxicity parallels grade 1 or 2 “maculopapular rash/dermatitis” as defined by CTCAE, since consensus recommendations from the Society for Immunotherapy of Cancer (SITC) Toxicity Management Working Group recommend systemic corticosteroids only for events higher than grade 2 [15].

The observed reduction in PFS benefit from ICI in severe ST compared with mild ST as defined in this study reinforces the importance of including clinical indications of immunosuppressive therapy in grading irAEs. The average highest dose of corticosteroids administered per treatment line for MM patients, which includes for management of non-skin irAEs, varied significantly between the three groups. It is expected that the severe ST group had the highest average dose administered, as systemic corticosteroid use was an inclusion criterion for this group. What is notable, however, is that the mild ST group had the best ICI outcomes and the lowest average dose, while the no ST group had the worst outcomes and the middle average dose. This suggests that developing ST reflects systemically heightened T cell function, which may necessitate treatment with corticosteroids that contributes to a reduction in clinical benefit from immunotherapy. Given that ICI success relies on the activation of cytotoxic T cells, this relatively diminished anti-tumor response might result from the anti-proliferative effects oral corticosteroids have on T cells [3, 16]. However, Additional file 1: Fig. S1 illustrates that patients in this analysis did not have different survival when stratified by no vs any use of systemic corticosteroids for ST, which challenges this argument. This relationship held true for each of the three systemic corticosteroids given (dexamethasone, prednisone, and methylprednisolone).

It is important to consider the possibility that patients who have longer survival are more likely to develop toxicity, simply because they have more time on treatment. To that end, the survivor bias analysis revealed the lack of association between development of severe ST and PFS, but not OS. Of note, patients with severe ST were significantly more likely to discontinue ICI due to toxicity, which may help account for this discrepancy in PFS. On the other hand, patients with no ST were more likely to discontinue ICI due to progression of disease, while patients with mild and severe ST had similar rates of discontinuation due to progression of disease. It is possible that severe ST patients had significant OS but not PFS benefit because OS may be influenced by treatments received after ICI. Any findings with groups formulated in the middle of survival time are prone to such bias, which emphasizes the importance of predictive biomarkers to optimize treatment selection and prophylactic care when indicated.

## Conclusions

The skin’s size and extensive immune cell network make it a prime target for ICI-induced toxicity, as well as a potential indicator for a systemically activated immune system. As such, we recommend considering how treatment of ST with systemic corticosteroids may reduce the efficacy of ICI, in addition to rigorously analyzing potential associations that are not explained with pre-clinical data. Moreover, we add the clinically relevant variable of systemic steroid use to prior ST grading systems, which allowed for a more robust assessment of ICI treatment outcomes based on ST severity. However, our data do not support that the use of systemic corticosteroids is the sole reason for survival differences, thus suggesting disease- and host-intrinsic factors at play. While future investigations on the mechanisms that link these two facets of immunotherapy are necessary, active clinical research should focus on baseline predictors of response and toxicity, for they overcome artificial survival advantages and provide more immediate clinical utility.

## Supplementary information

**Additional file 1: Figure S1.** Receiving systemic corticosteroids for skin toxicity (ST) does not lead to survival differences. **a** PFS and **b** OS by grouping of receiving systemic corticosteroids for ST vs not receiving systemic corticosteroids at all. All p-values are from the log-rank tests.

## Data Availability

Some of the data generated or analyzed during this study are included in this manuscript. All additional data can be made available from the corresponding author on reasonable request.

## Abbreviations

aHR: Adjusted hazard ratio
CTCAE: Common Terminology Criteria for Adverse Events
ECOG: Eastern Cooperative Oncology Group
HR: Hazard ratio
ICI: Immune checkpoint inhibition
irAEs: Immune-related adverse events
MM: Metastatic melanoma
ORR: Overall response rate
OS: Overall survival
PFS: Progression-free survival
RECIST: Response evaluation criteria in solid tumors
ST: Skin toxicity

## References

[1] Larkin J, Chiarion-Sileni V, Gonzalez R (2015). Combined Nivolumab and Ipilimumab or Monotherapy in Untreated Melanoma. N Engl J Med.

[2] Larkin J, Chiarion-Sileni V, Gonzalez R (2019). Five-Year Survival with Combined Nivolumab and Ipilimumab in Advanced Melanoma. N Engl J Med.

[3] Marin-Acevedo JA, Dholaria B, Soyano AE (2018). Next generation of immune checkpoint therapy in cancer: new developments and challenges. J Hematol Oncol.

[4] Donnelly D, Bajaj S, Yu J (2019). The complex relationship between body mass index and response to immune checkpoint inhibition in metastatic melanoma patients. J Immunother Cancer.

[5] Chat V, Ferguson R, Simpson D (2019). Autoimmune genetic risk variants as germline biomarkers of response to melanoma immune-checkpoint inhibition. Cancer Immunol Immunother.

[6] Gowen MF, Giles KM, Simpson D (2018). Baseline antibody profiles predict toxicity in melanoma patients treated with immune checkpoint inhibitors. J Transl Med.

[7] Peters BA, Wilson M, Moran U (2019). Relating the gut metagenome and metatranscriptome to immunotherapy responses in melanoma patients. Genome Med.

[8] Hryniewicki AT, Wang C, Shatsky RA (2018). Management of Immune Checkpoint Inhibitor Toxicities: a Review and Clinical Guideline for Emergency Physicians. J Emerg Med.

[9] Freeman-Keller M, Kim Y, Cronin H (2016). Nivolumab in resected and unresectable metastatic melanoma: characteristics of immune-related adverse events and association with outcomes. Clin Cancer Res.

[10] Rzepecki AK, Cheng H, McLellan BN (2018). Cutaneous toxicity as a predictive biomarker for clinical outcome in patients receiving anticancer therapy. J Am Acad Dermatol.

[11] Sanlorenzo M, Vujic I, Daud A (2015). Pembrolizumab cutaneous adverse events and their association with disease progression. JAMA Dermatol.

[12] Sibaud V (2018). Dermatologic reactions to immune checkpoint inhibitors : skin toxicities and immunotherapy. Am J Clin Dermatol.

[13] Wang Y, Zhou S, Yang F (2019). Treatment-related adverse events of PD-1 and PD-L1 inhibitors in clinical trials: a systematic review and meta-analysis. JAMA Oncol.

[14] Eggermont AMM, Kicinski M, Blank CU (2020). Association between immune-related adverse events and recurrence-free survival among patients with stage III melanoma randomized to receive pembrolizumab or placebo: a secondary analysis of a randomized clinical trial. JAMA Oncol.

[15] Puzanov I, Diab A, Abdallah K (2017). Managing toxicities associated with immune checkpoint inhibitors: consensus recommendations from the Society for Immunotherapy of Cancer (SITC) Toxicity Management Working Group. J Immunother Cancer.

[16] Pallet N, Fernández-Ramos AA, Loriot M-A (2018). Impact of immunosuppressive drugs on the metabolism of T cells. Int Rev Cell Mol Biol.

