# Supplementary figures and images for: Revisiting the association between skin toxicity and better response in advanced cancer patients treated with immune checkpoint inhibitors
Source: J Transl Med. 2020 Nov 11;18:430. doi: 10.1186/s12967-020-02612-5 (PMC7659132; doi:10.1186/s12967-020-02612-5)

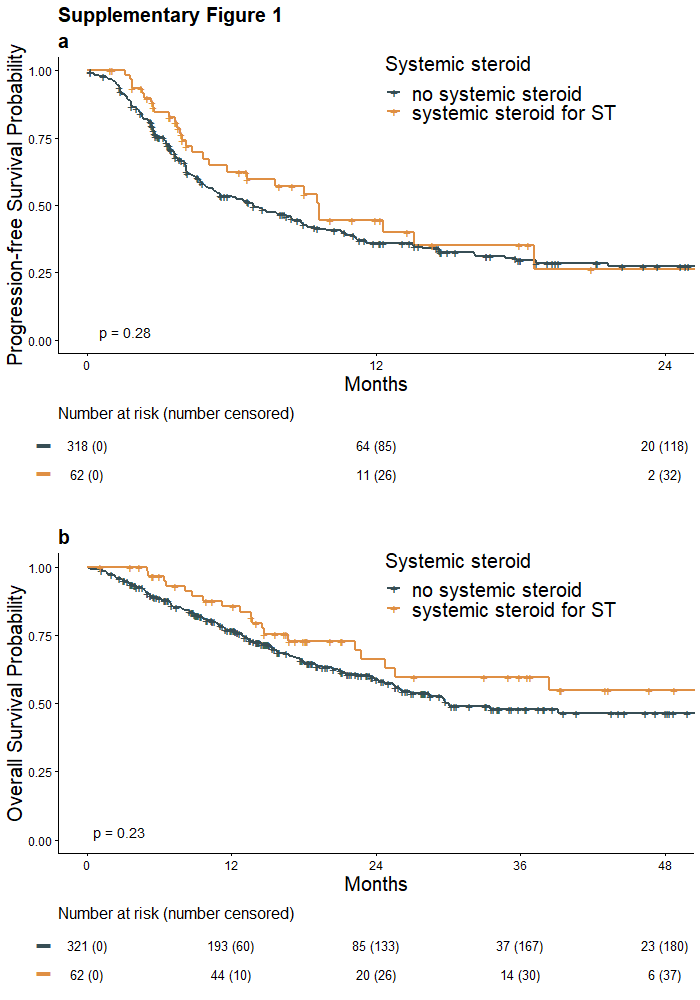

Supplement: Supplementary file 1 — Additional file 1: Figure S1. Receiving systemic corticosteroids for skin toxicity (ST) does not lead to survival differences. a PFS and b OS by grouping of receiving systemic corticosteroids for ST vs not receiving systemic corticosteroids at all. All p-values are from the log-rank tests. [file 12967_2020_2612_MOESM1_ESM.tif]
